# Wrist circumference as a novel predictor of transition from metabolically healthy to unhealthy phenotype in overweight/obese adults: a gender-stratified 15.5-year follow-up

**DOI:** 10.1186/s12889-021-12371-7

**Published:** 2021-12-13

**Authors:** Pouria Mousapour, Maryam Barzin, Majid Valizadeh, Maryam Mahdavi, Farzad Hadaegh, Fereidoun Azizi, Farhad Hosseinpanah

**Affiliations:** 1 Obesity Research Center, Research Institute for Endocrine Sciences, Shahid Beheshti University of Medical Sciences, Tehran, Iran; 2 Prevention of Metabolic Disorders Research Center, Research Institute for Endocrine Sciences, Shahid Beheshti University of Medical Sciences, Tehran, Iran; 3 Endocrine Research Center, Research Institute for Endocrine Sciences, Shahid Beheshti University of Medical Sciences, Tehran, Iran

**Keywords:** Wrist circumference, Metabolically healthy obesity, Metabolically unhealthy obesity, Transition, Gender

## Abstract

**Background:**

Individuals with transition from metabolically healthy overweight/obese (MHO) to metabolically unhealthy overweight/obese (MUO) phenotype are significantly predisposed to greater risks of cardiovascular events compared to those with a persistent MHO phenotype. The aim of this study was to evaluate the predictive performance of wrist circumference for this transition in adults over a 15.5-year follow-up.

**Methods:**

We included 309 males and 821 females with the age of ≥18 years old, body mass index ≥25 kg/m^2^, and metabolically healthy status according to the criteria of the Joint Interim Statement. The incidence of MUO phenotype was evaluated for each gender, across tertiles wrist circumference, using Cox-proportional hazard models.

**Results:**

The overall rate of transition from MHO to MUO phenotype was 87.1% in males and 77.5% in females. The hazard ratios (HRs) with 95% CI across second and third tertiles of wrist circumference were 0.89 (0.64-1.24) and 1.31 (0.99-1.73) in men (P for trend =0.027); and 1.34 (1.09-1.66) and 1.61 (1.30-2.00) in women (P for trend <0.001), respectively. After multivariable adjustment, HRs across second and third tertiles of wrist circumference were 0.92 (0.64-1.32) and 1.18 (0.83-1.67) in males (p for trend =0.352), and 1.32 (1.05-1.65) and 1.34 (1.06-1.96) in females (p for trend =0.025), respectively.

**Conclusions:**

Wrist circumference significantly predicts the transition from MHO to MUO phenotype in adults of both genders. However, it is an independent predictor of the transition only in females. Future studies are warranted to clarify the role of wrist circumference mechanisms on metabolic risk deterioration.

## Introduction

The prevalence of obesity is rising worldwide [1]. Obesity is regarded as an independent risk factor for a broad spectrum of non-communicable and cardiovascular diseases (CVDs) and a leading but preventable cause of reduced life expectancy and quality of life [2]. It is well established that individuals in the same body mass index (BMI) category can display substantial heterogeneity regarding typical metabolic disorders associated with obesity, and metabolically healthy overweight/obese (MHO) phenotype is a description of a subgroup of overweight or obese individuals with normal glucose tolerance, blood pressure, lipid profile, and waist circumference. The prevalence of MHO not only is strongly dependent on age, gender, and ethnic disparities of different populations, but it also greatly varies according to different definition criteria of metabolic health [3]. A 2017 meta-analysis estimated the overall prevalence of MHO in obese individuals as 35%, ranging between 13% and 86% [4].

Nevertheless, not all individuals with MHO phenotype follow the same natural history. An extensive body of literature supports that MHO can be a transient condition, and approximately 30 to 50% of individuals with MHO have shown transition to metabolically unhealthy overweight/obese (MUO) phenotype during 4 to 20 years of follow-up [5]. Compelling evidence supports that in comparison with metabolically healthy normal weight individuals, those with MHO phenotype are at an increased risk of CVDs but only when they are followed up for at least 10 years [6, 7]. Moreover, MHO individuals at transition to MUO are significantly predisposed to greater risks of CVDs compared to those with persistent MHO phenotype [8–10]. Although the inconsistency in the prognosis of MHO phenotype could be explained by short-term follow-up periods, such studies probably fail to detect all individuals who had MUO transition.

Several anthropometric indices have been proposed as predictors of obesity-related cardiometabolic complications. Wrist circumference is an inexpensive and easily available measure of skeletal frame size and also a surrogate for estimating peripheral fat distribution. Wrist circumference has been shown to be associated with high blood pressure, kidney dysfunction, low HDL, abdominal obesity, MUO phenotype and cardiovascular risk in children and adolescents [11–15]. It has also been suggested to be positively associated with hypertension, insulin resistance and diabetes mellitus, metabolic syndrome and cardiovascular disease among the adult population [16–21]. A recent meta-analysis of available evidence indicated that greater wrist circumference was associated with increased incidence of metabolic syndrome in adults, albeit with high between-studies heterogeneity that was mainly related to gender [22].

No study has investigated the predictive performance of wrist circumference for transition from MHO to MUO phenotype. The aim of this study was to evaluate the association between wrist circumference and the incidence of MUO phenotype in a gender-stratified 15.5-year follow-up study on Iranian adults with MHO.

## Materials and methods

### Study design and participants

Tehran Lipid and Glucose Study (TLGS) is an observational population-based ongoing cohort which commenced in February 1999, aiming to determine the risk factors of non-communicable diseases in a representative population of urban residents of Tehran, Iran. The rationale and design of the TLGS have been published elsewhere [23]. Recruitment of participants was conducted by the multistage cluster random sampling technique from the urban district 13 of Tehran (phase I from 1999 to 2001 and phase II from 2002 to 2005). All participants completed baseline interviews, anthropometric examinations, and laboratory measurements. Follow-up assessments were performed in subsequent surveys at approximately 3.6-year intervals, as phase III (2006–2008), phase IV (2009–2011), phase V (2012-2015), and phase VI (2016-2018).

We initially included the baseline population from phases I and II. From a total 12682 individuals aged ≥18 years, 2519 subjects with BMI ≥25 kg/m^2^ met the criteria of MHO phenotype. We then excluded 53 individuals with histories of any cancer, 247 because of using systemic corticosteroids, 106 for pregnancy at any follow-up point, and 289 for loss to follow-up, leaving an ultimate valid sample of 1130 for analysis.

This study was conducted in accordance with the 1964 Helsinki declaration and its later amendments, and all the procedures involving study participants were approved by the National Research Council of the Islamic Republic of Iran (No. 121), the Human Research Review Committee of the Endocrine Research Center, Shahid Beheshti University, Tehran, Iran. All participants provided written informed consent.

### Anthropometrics and biochemical measurements

Data of the TLGS program including interviews, anthropometrics, and biochemical measurements were recorded and collected by trained general practitioners. Information regarding histories of diabetes and cardiovascular diseases, drug use, physical activity, smoking habits, and educational level was obtained through pretested interview questionnaires. Details on anthropometric measurements including height, weight, waist circumference (WC), and hip circumference are available elsewhere [23]. For measuring wrist circumference, subjects were asked to hold their wrist anterior surface using a tape meter up to the nearest 0.1 cm. Without any pressure, the superior border of the tape measure was placed just distal to the prominence of radial and ulnar bones. Information about physical activity was collected using the Persian-translated forms of Lipid Research Clinic (LRC) questionnaire in phase I [24]. Because the precision of the LRC was not reliable, we used the Modifiable Activity Questionnaire (MAQ) which uses all types of activity including leisure time, household and job activities, in the remaining follow-up examinations [23].

Venous blood samples were obtained from all participants between 07:00 and 09:00 a.m. after 12 to 14 hours of overnight fasting and analyzed on the same day of sampling. Detailed laboratory protocols of TLGS have been reported previously [23]. Plasma total cholesterol (TC) and triglyceride (TG) levels were assayed using enzymatic colorimetric methods with cholesterol esterase/cholesterol oxidase and glycerol phosphate oxidase, respectively. High-density lipoprotein (HDL) was measured after precipitating the apolipoprotein B containing lipoproteins with phosphotungstic acid. Low-density lipoprotein (LDL) was calculated using the Friedwald formula. FPG and 2 h-PG were measured by an enzymatic colorimetric method, using glucose oxidase technique.

### Definitions

The participants were categorized by their smoking status as: 1. smokers (currently using any tobacco product either daily or occasionally) and 2. non-smokers (ex-smokers or those who have never smoked). Since duration of physical activity was not included in the LRC, participants who were registered in the study from the first examination of TLGS, were considered to have high physical activity if participating in robust physical activity for minimum of 3 days per week. Individuals who enrolled in the study at the second phase of the TLGS, were considered to have high physical activity if they achieved a minimum of at least 600 MET (metabolic equivalent task)-minutes per week [25]. BMI was calculated as weight (Kg) divided by square of height (meter). Waist-to-hip ratio (WHR) was calculated as WC divided by the hip circumference; and waist-to-height ratio (WHtR) was calculated as WC (cm) divided by height (cm).

Metabolic health components were defined using the criteria proposed by the Joint Interim Statement (JIS) [26] as follows: (1) FBS ≥100 mg/dl (5.6 mmol/l) or 2-h blood glucose ≥140 mg/dl (7.8 mmol/l) or drug treatment; (2) fasting TGs ≥150 mg/dl (1.7 mmol/l) or drug treatment for dyslipidemia; (3) fasting HDL-C <50 mg/dl (1.29 mmol/l) in women and <40 mg/dl (1.03 mmol/l) in men or drug treatment; (4) systolic blood pressure (SBP)≥ 130 mmHg, diastolic blood pressure (DBP)≥ 85 mmHg or antihypertensive drug treatment; and (5) WC ≥89 cm in men and ≥91 cm in women based on appropriate cutoff points of WC for predicting the incidence of CVDs among Iranian men and women [27]. Participants with BMI ≥25 kg/m^2^ were considered overweight/obese. MHO phenotype was defined as being overweight/obese with null or one of the JIS metabolic components, and MUO phenotype was defined as being overweight/obese subjects with two or more of the JIS metabolic components.

### Statistical analysis

Baseline characteristics were expressed as mean ± standard deviation for normally distributed variables, median (interquartile 25-75% [IQ1-3]) for skewed variables, or frequency (percentage) for categorical variables. Interaction between wrist circumference and gender at transition from MHO to MUO phenotype was tested by the log-likelihood ratio test, and since we found a significant interaction (P =0.037), the analyses were performed in males and females, separately. Baseline comparisons were performed across the tertiles of wrist circumference using one-way analysis of variance (ANOVA) and Mann–Whitney U tests for continuous variables and the Chi Square test for categorical variables. Survival time was considered from the start of the follow-up period to the date of first transition to MUO phenotype. The censoring time of an individual was the time from entry into the study to either the loss to follow-up point, death from any cause, or the end of the study without having the transition, whichever happened first. Kaplan-Meier survival curves were plotted to demonstrate MUO transition within tertiles of wrist circumference. The Log-Rank test was performed to assess the significance of trends across tertiles of wrist circumference. Using Cox-proportional hazards models, transition from MHO to MUO phenotype (expressed per 1000 person-years) was tested across tertiles of wrist circumference, separately for each gender, with and without adjustments for potential confounders. Proportional hazards assumptions in the Cox model were checked graphically using the Schoenfeld’s test of residuals [28]; all proportionality assumptions were generally appropriate. Considering wrist circumference as a continuous variable, acquired hazard ratios were also assessed for each SD increment in wrist circumference, separately for each gender. All calculations were performed in SPSS software version 24.0 (SPSS Inc, Chicago, IL, USA). A two-tailed P value of ≤0.05 was considered as the statistical significance threshold.

## Results

A total of 309 males and 821 females with MHO phenotype were included. The mean age of the total study population was 35.68 ± 11.44 years, and males and females were comparable for age. Comparison of baseline characteristics between genders has been presented in Table 1. At baseline, the ratio of smokers, SBP, and serum FPG and triglycerides were higher, and serum HDL level was lower in males compared to females while no significant differences were seen regarding physical activity and serum levels of TC and LDL between genders. The mean age of males and females were similar at the time of transition from MHO to MUO phenotype. At this time, the prevalence of smoking, mean SBP, DBP and FPG were higher, while the prevalence of low physical activity, and mean serum levels of total cholesterol, HDL, 2-h PG were lower in males compared to females. There was no significant difference regarding the prevalence of anti-hypertension and anti-diabetes drug therapy at the time of transition between genders, while anti-dyslipidemia drug therapy was more prevalent among females compared to males. At the study baseline and the time of transition, males had lower mean BMI, hip circumference and WHtR, but higher WC, WHR, and wrist circumference in comparison with females.

**Table 1:** Comparison of characteristics between males and females at the study baseline and the time of transition from metabolically healthy to metabolically unhealthy overweight/obese phenotype

Variables	Baseline characteristics			Characteristics at transition		
	Males (*n*=309)	Females (*n*=821)	*P* value	Males (*n*=269)	Females (*n*=636)	*p*-value
Wrist circumference, cm	17.80 ± 0.77	15.89 ± 0.77	<0.001	18.17 ± 0.86	15.87 ± 1.02	<0.001
Age, years	36.42 ± 12.58	35.40 ± 10.98	0.208	43.50 ± 12.67	44.01 ± 11.20	0.572
Smokers, n (%)	73 (23.8)	22 (2.7)	<0.001	48 (17.8)	11 (1.7)	<0.001
Low physical activity, n (%)	210 (68.9)	518 (63.6)	0.098	156 (58.6)	469 (74.4)	<0.001
SBP, mmHg	112.69 ± 9.92	109.47 ± 11.38	<0.001	117.11 ± 13.20	112.54 ± 14.91	<0.001
DBP, mmHg	73.22 ± 7.15	73.21 ± 8.14	0.987	76.90 ± 8.98	75.33 ± 9.77	0.024
FPG, mg/dl	88.47 ± 8.18	86.45 ± 10.01	0.002	94.35 ± 13.90	91.63 ± 13.66	0.006
2-h PG, mg/dl	97.62 ± 31.64	101.43 ± 25.24	0.044	102.41 ± 39.56	110.16 ± 32.97	0.003
Triglycerides^a^, mg/dl	108.00 (86.00-133.00)	96.00 (75.00-121.00)	<0.001	127.00 (91.00-167.00)	115.00 (85.00-160.00)	0.141
Total cholesterol, mg/dL	193.77 ± 36.63	195.77 ± 38.57	0.431	188.00 ± 33.36	195.90 ± 36.68	0.002
HDL, mg/dL	43.55 ± 8.24	49.84 ± 11.85	<0.001	41.16 ± 11.45	47.38 ± 11.90	<0.001
LDL, mg/dL	128.03 ± 32.87	126.21 ± 33.71	0.417	120.15 ± 29.51	122.17 ± 31.96	0.382
Anti-hypertension drug therapy, n (%)	1 (0.3)	7 (0.9)	0.334	7 (2.6)	27 (4.1)	0.334
Anti-diabetes drug therapy, n (%)	1 (0.3)	0 (0)	0.103	2 (0.7)	11 (1.7)	0.364
Anti-dyslipidemia drug therapy, n (%)	0 (0)	0 (0)	N/A	7 (2.6)	44 (7.7)	0.011
BMI, kg/m^2^	27.14 ± 2.16	27.94 ± 2.59	<0.001	28.86 ± 2.72	30.56 ± 3.25	0.001
BMI 25-29.9 kg/m^2^, n (%)	283 (91.6)	683 (83.2)	<0.001	203 (75.5)	330 (51.9)	<0.001
BMI ≥30 kg/m^2^, n (%)	26 (8.4)	138 (16.8)		66 (24.5)	306 (48.1)	
WC, cm	90.06 ± 7.58	84.36 ± 7.46	<0.001	99.19 ± 7.19	95.44 ± 7.82	<0.001
Hip circumference, cm	99.10 ± 4.83	105.20 ± 6.30	<0.001	102.79 ± 5.69	107.17 ± 7.21	<0.001
WHR	0.91 ± 0.06	0.80 ± 0.06	<0.001	0.96 ± 0.05	0.89 ± 0.07	<0.001
WHtR	0.53 ± 0.05	0.56 ± 0.05	<0.001	0.58 ± 0.04	0.61 ± 0.05	<0.001

Categorical variables are represented as frequency (percent). Continuous variables are represented as mean ± SD

*SBP* systolic blood pressure, *DBP* diastolic blood pressure, *FPG* fasting plasma glucose, *2-h PG* 2-h post-challenge plasma glucose, *HDL* high-density lipoprotein, *LDL* low-density lipoprotein, *BMI* body mass index, *WC* waist circumference, *WHR* waist-to-hip ratio, *N/A* not applicable

^a^Triglycerides is reported as median (IQR 25-75)

The baseline characteristics of male and female participants across tertiles of wrist circumference have been illustrated in Table 2. Age significantly increased across the tertiles of wrist circumference in both genders. The prevalence of smoking and low physical activity was not associated with wrist circumference. Laboratory measures were not associated with increased wrist circumference except for FPG and HDL in males and SBP in females. On the other hand, mean weight, height, BMI, WC, hip circumference, WHR and WHtR increased across baseline wrist circumference tertiles in both males and females.

**Table 2:** Baseline characteristics of males and females across tertiles of wrist circumference

	Males				Females			
Variables	Tertile 1 (< 17.5 cm)	Tertile 2 (17.5-18.0 cm)	Tertile 3 (18.0 cm <)	*P* for trend	Tertile 1 (< 15.5 cm)	Tertile 2 (15.5-16.1 cm)	Tertile 3 (16.1 cm <)	*P* for trend
Wrist circumference, cm	16.95 ± 0.40	17.64 ± 0.14	18.48 ± 0.49	<0.001	14.94 ± 0.43	15.76 ± 0.22	16.71 ± 0.48	<0.001
Age, years	32.69 ± 9.43	34.62 ± 11.54	40.02 ± 14.07	<0.001	34.10 ± 9.67	34.24 ± 10.73	37.73 ± 11.77	<0.001
Smokers, n (%)	27 (28.4)	13 (17.1)	33 (24.3)	0.221	8 (4.1)	10 (2.9)	4 (1.4)	0.192
Low physical activity, n (%)	66 (71.0)	53 (69.7)	91 (66.9)	0.794	119 (62.0)	225 (65.4)	174 (62.4)	0.642
SBP, mmHg	111.24 ± 10.05	112.25 ± 9.88	113.94 ± 9.77	0.114	107.60 ± 11.00	109.19 ± 10.71	111.12 ± 12.21	0.003
DBP, mmHg	73.17 ± 6.60	72.13 ± 6.97	73.87 ± 7.58	0.230	72.18 ± 7.90	73.50 ± 8.37	73.57 ± 7.97	0.130
FPG, mg/dl	87.57 ± 7.74	87.17 ± 6.20	89.82 ± 9.24	0.033	85.67 ± 6.99	86.12 ± 7.02	87.39 ± 14.07	0.135
2-h PG, mg/dl	95.15 ± 25.60	97.13 ± 24.54	99.60 ± 38.24	0.594	99.78 ± 24.78	100.86 ± 23.91	103.27 ± 27.05	0.312
Triglycerides^a^, mg/dl	114.00 (87.00-142.00)	112.00 (82.00-133.50)	103.00 (84.50-129.50)	0.103	92.00 (72.00-120.00)	95.00 (75.00-121.00)	98.50 (78.25-122.00)	0.329
Total cholesterol, mg/dL	195.07 ± 32.39	196.05 ± 41.33	191.58 ± 36.74	0.636	191.50 ± 36.91	196.70 ± 39.96	197.56 ± 37.86	0.203
HDL, mg/dL	41.38 ± 9.09	43.84 ± 8.80	44.89 ± 6.95	0.005	48.81 ± 11.32	50.32 ± 12.18	49.95 ± 11.78	0.360
LDL, mg/dL	130.33 ± 27.39	129.88 ± 37.87	125.37 ± 33.44	0.453	122.95 ± 32.55	126.83 ± 35.04	127.65 ± 32.75	0.303
Weight. Kg	74.13 ± 5.66	78.00 ± 6.33	82.57 ± 9.17	<0.001	64.73 ± 5.50	67.90 ± 6.26	72.93 ± 7.83	<0.001
Height, cm	167.96 ± 5.72	170.25 ± 5.60	172.04 ± 6.56	<0.001	154.56 ± 5.40	156.74 ± 5.26	158.98 ± 6.14	<0.001
BMI, kg/m^2^	26.26 ± 1.15	26.90 ± 1.61	27.89 ± 2.66	<0.001	27.9 ± 1.77	27.65 ± 2.32	28.88 ± 3.07	<0.001
WC, cm	86.54 ± 5.19	89.44 ± 5.56	92.86 ± 8.80	<0.001	81.68 ± 6.09	83.76 ± 6.69	86.96 ± 8.38	<0.001
Hip circumference, cm	96.93 ± 4.02	98.97 ± 3.54	100.68 ± 5.37	<0.001	103.49 ± 5.54	104.60 ± 5.87	107.14 ± 6.81	<0.001
WHR	0.89 ± 0.05	0.90 ± 0.05	0.92 ± 0.67	0.001	0.79 ± 0.06	0.80 ± 0.06	0.81 ± 0.62	0.001
WHtR	0.51 ± 0.03	0.53 ± 0.04	0.54 ± 0.06	<0.001	0.53 ± 0.05	0.53 ± 0.05	0.55 ± 0.05	<0.001

Categorical variables are represented as frequency (percent). Continuous variables are represented as mean ± SD

*SBP* systolic blood pressure, *DBP* diastolic blood pressure, *FPG* fasting plasma glucose, *2-h PG* 2-h post-challenge plasma glucose, *HDL* high-density lipoprotein, *LDL* low-density lipoprotein, *BMI* body mass index, *WC* waist circumference, *WHR* waist-to-hip ratio

^a^Triglycerides is reported as median (IQR 25-75)

**Fig. 1:**
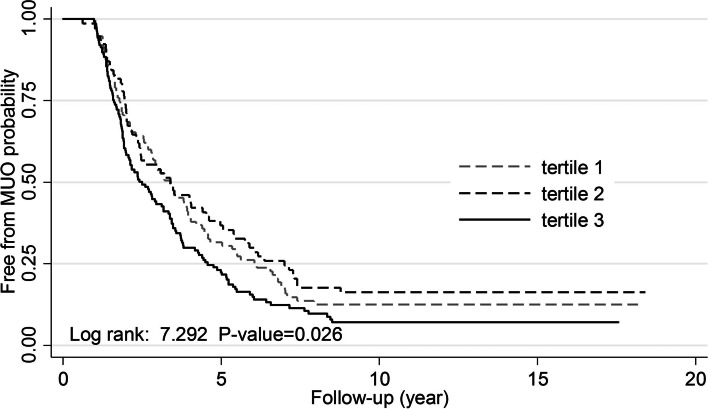
Kaplan-Meier curves for transition from MHO to MUO phenotype across tertiles of wrist circumference in males

**Fig. 2:**
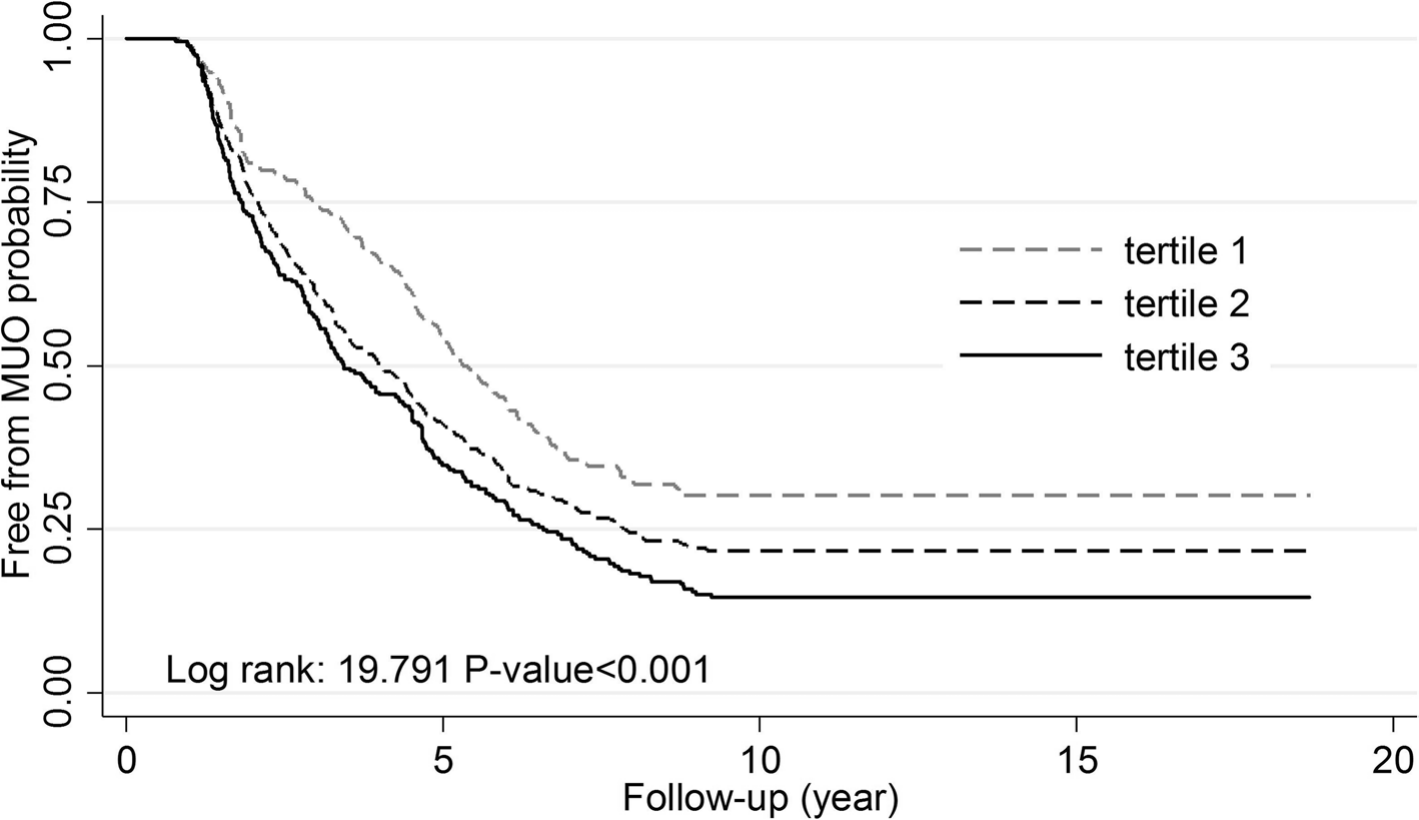
Kaplan-Meier curves for transition from MHO to MUO phenotype across tertiles of wrist circumference in females

Transition from MHO to MUO phenotype occurred at the median of 2.5 (IQ: 1.6-4.3) and 3.2 (IQ: 1.8-5.1) years of follow-up with the last transition at 8.8 and 9.2 years in males and females, respectively. Mean ages of males and females at the time of transition were 43.5 ± 12.7 and 44.0 ± 11.2 years, respectively. Figures 1 and 2 represent gender-specific Kaplan-Meier curves for MHO to MUO transition across the tertiles of wrist circumference. Among males and females, the probability of maintaining a MHO phenotype significantly decreased in a stepwise fashion across wrist circumference tertiles (P for trend =0.043 and <0.001, respectively).

Over a median of 15.5 (IQ1-3: 12.5-16.6) years of follow-up, MUO phenotype was identified in 269 (87.1%) males and 636 (77.5%) females. The rates of MHO to MUO transition across subsequent wrist circumference tertiles were 86.3%, 81.82%, and 90.51% among males and 69.7%, 76.66%, and 84.28% among females. Over 6,068.30 person-years of follow-up, the rate MHO to MUO transition per 1000 person-years across the first, second, and third tertiles of wrist circumference were 187.73 (151.19-233.10), 163.25 (127.53-208.98), and 245.25 (205.67-292.45) in males, and 101.16 (85.40-119.82), 131.75 (116.83-148.57), and 169.00 (148.76-192.00) in females, respectively.

As demonstrated in Tables 3 and 4, the hazard ratios (HRs, with 95% CI) of transition to the MUO phenotype across the second and third tertiles of wrist circumference were 0.89 (0.64-1.24) and 1.31 (0.99-1.73) in men (P for trend =0.027) and 1.34 (1.09-1.66) and 1.61 (1.30-2.00) in women (P for trend <0.001), respectively. After multivariable adjustments, HRs across the second and third tertiles of wrist circumference were 0.92 (0.64-1.32) and 1.18 (0.83-1.67) in males (P for trend =0.352) and 1.32 (1.05-1.65) and 1.34 (1.06-1.96) in females (P for trend =0.025), respectively. In males, age (HR: 1.28 [1.08-1.50], *P*-value: 0.004), FPG (HR: 1.24 [1.03-1.48], *P*-value: 0.019), HDL (HR: 0.73 [0.61-0.88], *P*-value: 0.001) and BMI (HR: 1.36 [1.07-1.73], *P*-value: 0.013) were independently associated with transition from MHO to MUO transition; while in females, apart from wrist circumference, age (HR: 1.38 [1.25-1.52], P-value: <0.001), FPG (HR: 1.14 [1.02-1.28], P-value: 0.023), Triglycerides (HR: 1.18 [1.10-1.27], P-value: <0.001), HDL (HR: 0.77 [0.70-0.84], P-value: <0.001) and waist circumference (HR: 1.17 [1.05-1.31], P-value: 0.005) were independently associated with the risk of future MHO to MUO transition.

**Table 3:** Hazard ratios (HRs) and 95% confidence intervals (CI) for transition from MHO to MUO phenotype based on tertiles of wrist circumference in males

	Unadjusted		Age-adjusted		Model 1			Model 2	
	HR (%95 CI)	***P*** -value	HR (%95 CI)	***P*** -value	HR (%95 CI)		***P*** -value	HR (%95 CI)	***P*** -value
Wrist circumference									
Tertile 1 (<17.5 cm)	1.00 (reference)		1.00 (reference)		1.00 (reference)			1.00 (reference)	
Tertile 2 (17.5-18.0 cm)	0.89 (0.64-1.24)	0.489	0.86 (0.62-1.20)	0.377	0.89 (0.63-1.27)		0.522	0.92 (0.64-1.32)	0.661
Tertile 3 (>18.0 cm)	1.31 (0.99-1.73)	0.060	1.16 (0.87-1.55)	0.314	1.15 (0.84-1.59)		0.372	1.18 (0.83-1.67)	0.352
*P* for trend	**0.027**		0.165		0.306			0.352	
Age			**1.26 (1.12-1.39)**	**0.001**	**1.24 (1.07-1.43)**		**0.004**	**1.28 (1.08-1.50)**	**0.004**
Smoking					1.07 (0.79-1.45)		0.667	1.08 (0.79-1.46)	0.634
Education level (below diploma)					1.18 (0.86-1.61)		0.308	1.18 (0.86-1.61)	0.313
Low physical activity					1.16 (0.88-1.54)		0.293	1.13 (0.85-1.50)	0.395
SBP					0.97 (0.82-1.14)		0.714	0.98 (0.83-1.16)	0.799
DBP					1.09 (0.93-1.29)		0.294	1.08 (0.92-1.28)	0.343
FPG					**1.22 (1.02-1.46)**		**0.028**	**1.24 (1.03-1.48)**	**0.019**
2-h PG					1.03 (0.90-1.18)		0.670	1.01 (0.88-1.16)	0.832
Triglycerides					1.09 (0.97-1.23)		0.153	1.10 (0.97-1.24)	0.124
HDL					**0.75 (0.63-0.90)**		**0.001**	**0.73 (0.61-0.88)**	**0.001**
Body mass index								**1.36 (1.07-1.73)**	**0.013**
Waist circumference								0.85 (0.66-1.08)	0.179
Hip circumference								0.91 (0.68-1.21)	0.510

Hazard ratios for the continuous variables are reported per 1 standard deviation

*SBP* systolic blood pressure, *DBP* diastolic blood pressure, *FPG* fasting plasma glucose, *2-h PG* 2-h post-challenge plasma glucose, *HDL* high-density lipoprotein, *LDL* low-density lipoprotein

**Table 4:** Hazard ratios (HRs) and 95% confidence intervals (CI) for transition from MHO to MUO phenotype based on tertiles of wrist circumference in females

	Unadjusted		Age-adjusted		Model 1			Model 2	
	HR (%95 CI)	***P*** -value	HR (%95 CI)	***P*** -value	HR (%95 CI)		***P*** -value	HR (%95 CI)	***P*** -value
Wrist circumference									
Tertile 1 (<15.5 cm)	1 (reference)		1 (reference)		1 (reference)			1 (reference)	
Tertile 2 (15.5-16.1 cm)	**1.34 (1.09-1.66)**	**0.005**	**1.35 (1.10-1.67)**	**0.004**	**1.33 (1.09-1.70)**		**0.007**	**1.32 (1.05-1.65)**	**0.012**
Tertile 3 (>16.1 cm)	**1.61 (1.30-2.00)**	**<0.001**	**1.50 (1.21-1.85)**	**<0.001**	**1.46 (1.16-1.84)**		**0.001**	**1.34 (1.06-1.96)**	**0.016**
*P* for trend	**<0.001**		**0.001**		**0.003**			**0.025**	
Age			**1.43 (1.31-1.56)**	**<0.001**	**1.43 (1.30-1.57)**		**<0.001**	**1.38 (1.25-1.52)**	**<0.001**
Smoking					1.17 (0.73-1.86)		0.512	1.21 (0.76-1.93)	0.420
Education level (below diploma)					0.93 (0.72-1.20)		0.575	0.91 (0.70-1.17)	0.456
Low physical activity					0.96 (0.81-1.14)		0.632	0.95 (0.70-1.13)	0.567
SBP					1.04 (0.94-1.05)		0.412	1.06 (0.96-1.17)	0.229
DBP					**1.12 (1.01-1.24)**		**0.038**	1.11 (0.99-1.23)	0.056
FPG					**1.17 (1.04-1.30)**		**0.007**	**1.14 (1.02-1.28)**	**0.023**
2-h PG					1.13 (0.92-1.10)		0.790	1.02 (0.93-1.11)	0.735
Triglycerides					**1.20 (1.11-1.29)**		**<0.001**	**1.18 (1.10-1.27)**	**<0.001**
HDL					**0.79 (0.72-0.87)**		**<0.001**	**0.77 (0.70-0.84)**	**<0.001**
Body mass index								1.02 (0.91-1.15)	0.691
Waist circumference								**1.17 (1.05-1.31)**	**0.005**
Hip circumference								0.98 (0.87-1.10)	0.715

Hazard ratios for the continuous variables are reported per 1 standard deviation

*SBP* systolic blood pressure, *DBP* diastolic blood pressure, *FPG* fasting plasma glucose, *2-h PG* 2-h post-challenge plasma glucose, *HDL* high-density lipoprotein, *LDL* low-density lipoprotein

The unadjusted HRs of MHO to MUO transition for each SD increment in wrist circumference (0.77 cm in both genders) were 1.25 (1.04-1.51) in males and 1.27 (1.14-1.42) in females. The age- and multivariable- (model 2) adjusted HRs of MHO to MUO transition for each SD increment in wrist circumference were 1.15 (0.95-1.40) and 1.22 (0.96-1.56) in males and 1.21 (1.08-1.35) and 1.13 (1.00-1.27) in females, respectively.

## Discussion

This is the first study demonstrating a positive association between increment in wrist circumference and the risk of transition from MHO to MUO phenotype in adult males and females. In our studied population; however, wrist circumference was not an independent predictor of MHO to MUO transition in males while each 0.77 cm increase in wrist circumference was independently associated with a 13% higher risk of the transition in females.

The MUO phenotype, defined as the coexistence of several metabolic abnormalities and adiposity, has a complex pathogenesis involving interactions among environmental, behavioral, and genetic factors. Although individuals with MHO have minimal metabolic abnormalities, potential factors can contribute to positive energy balance, adipogenesis, abnormal body fat distribution, and insulin resistance which play central roles in the development of MUO. Nevertheless, there is limited data available regarding the major predictors of this transition in MHO individuals. In a 10-year follow-up of 85 Japanese Americans with MHO, female gender, baseline HDL, fasting plasma insulin, and abdominal visceral fat, but not BMI, WC, LDL, TG, and thigh and abdominal subcutaneous fat were independent risk factors of MHO to MUO transition [29]. Moreover, gender-stratified 41-month follow-up of 2204 Korean adults with MHO phenotype demonstrated that menopause was an independent predictor of the metabolic transition in females [30]. In these studies, daily energy and macronutrients intake, alcohol consumption, and physical activity were not determinants of transition from MHO to MUO phenotype [29, 30]. Also, in a study with over 10 years of follow-up of metabolically healthy Tehranian adults with abdominal obesity, low serum HDL, hypertriglyceridemia, and insulin resistance were independent predictors of the transition to metabolically unhealthy phenotype [31]. Moreover, many studies have demonstrated associations between different genetic variants, cardiometabolic risk factors, and MUO phenotype [32–34]. Interestingly, a recently published article demonstrated that three genetic markers had contributing or protective effects on the transition from MHO to MUO phenotype, suggesting a gender-specific contribution for some genetic variants in the cardiometabolic deterioration of MHO individuals [35].

Wrist circumference is considered as a unique fat distribution marker. Supporting the association between adiposity and wrist circumference, a cross-sectional study in Italy by Maddaloni et al. demonstrated that wrist circumference was independently associated with visceral adiposity index and metabolic syndrome in Italian overweight/obese adults of both genders, but not in those with BMI <25 kg/m^2^ [18]. Evidence shows that compared to MUO, MHO is associated with more favorable proinflammatory and adipokine secretion profiles [5], which is supported by the observations implying a greater risk of cardiometabolic abnormalities in MUO individuals [36–38]. However, scarce data exists on the proinflammatory contribution of wrist adiposity in metabolic dysfunction. In a recent cross-sectional study in Italy on 280 children aged 7-18 years old, Luordi *et al*. showed that independent of age and gender, wrist circumference had the strongest negative correlation with adiponectin/leptin ratio as compared to total and truncal fat mass measured by dual-energy X-ray absorptiometry and was the only index correlating with CVD risk score, suggesting wrist circumference, and not total and truncal fat percentages, as an independent predictor of metabolic disturbances [14]. Similar to our observation among MHO adults, Amini et al. in a study of 1709 diabetic patients showed a significantly positive association of wrist circumference with BMI and WC, but a significantly inverse with HDL levels [39]. In our analysis, despite its significant correlations with BMI, WC, and hip circumference, wrist circumference still remained a dominant predictor of MHO to MUO transition independent of these surrogates for adipose tissue dysfunction [40].

Wrist circumference has also been suggested to be an easy and well-known index of skeletal frame size, and also a good surrogate to assess bone metabolism in relation to hyperinsulinemia, since insulin-like growth factor 1 (IGF 1) levels are major determinants of bone geometry. Capizzi et al. provided the first evidence showing that the transversal wrist internal bone tissue area determined by nuclear magnetic resonance, but not wrist circumference, was associated with fasting plasma insulin levels and insulin resistance in overweight/obese children and adolescents, suggesting the area of the wrist bone, and not wrist fat component as the main contributor to the relationship between wrist circumference and insulin resistance in children and adolescents [41]. However, considering the rapid skeletal growth in children and adolescents, and the anabolic effects of insulin on bone formation, by stimulating osteoblastic proliferation and inhibiting osteoclastic proliferation [42], these cross-sectional findings may not be generalizable for the adult population due to the cessation of skeletal growth after puberty. Moreover, evidence shows that the skeleton exerts an endocrine regulation by increasing production of insulin and improving glucose tolerance, increasing expression of adiponectin and reducing visceral fat [43, 44]. Hence, the potential association between wrist bone tissue and cardiometabolic deterioration in adults remains an open question for future investigations.

We also observed that after controlling all traditional cardiometabolic risk factors, increments in wrist circumference could still predict transition from MHO to MUO phenotype in females but not in males. This gender-disparity can be at least in part explained by the greater proportion of lean tissue in males’ wrist, as the same amount of fat deposition in females’ wrist causes a greater increment in their wrist circumference compared to males, and in other words, increasing wrist circumference is more associated with progression of adiposity-related metabolic effects in females. Nevertheless, this hypothesis requires further confirmation by magnetic resonance imaging studies. Furthermore, recent evidence implies a negative association between body lean mass and cardiovascular and metabolic diseases [45–47], that can potentially dilute the association of wrist adiposity with cardiometabolic deterioration in males. In support, a 9-year follow-up of Tehranian general male population demonstrated that, after adjustment for BMI or WC, increment in wrist circumference had a negative association with the incidence of cardiovascular events [48].

To the best of our knowledge, this is the first prospective study to assess the performance of wrist circumference for predicting the natural history of MHO adults. The population-based design and long term follow-up of a well-defined representative cohort are other noteworthy strengths of our study. However, the study has some limitations. First of all, we used the information collected only once at the study baseline and therefore did not apply possible changes in potential risk factors throughout the follow-up. Second, the LRC questionnaire used for collecting physical activity data in phase1 has not been validated in Iran. Third, due to unavailability of data for some participants, we did not include insulin resistance, as well as nutritional and socioeconomic status in our analysis. Another limitation is low number of male participants, which may decrease the statistical power. Last but not least, we did not validate the amount of different wrist components using gold-standard imaging modalities.

## Conclusions

In a long-term follow-up of a representative sample of Tehranian adult population, we observed that a relatively high proportion of MHO individuals progressed to MUO phenotype within about 10 years. This study found that wrist circumference significantly predicted MHO to MUO transition in adult males and females; however, it remained an independent predictor of the transition only in females. Wrist circumference could be considered as a novel and effortless anthropometric screening tool for predicting MHO to MUO transition in adults.

## Data Availability

The datasets used and analyzed during the current study available from the corresponding author on reasonable request.

## Abbreviations

MHO: Metabolically healthy overweight/obese
MUO: Metabolically unhealthy overweight/obese
BMI: Body mass index
CVD: Cardiovascular disease
TLGS: Tehran lipid and glucose study
WC: Waist circumference
WHtR: Waist to height ratio
WHR: Waist to hip ratio
BMI: Body mass index
HTN: Hypertension
SBP: Systolic blood pressure
DBP: Diastolic blood pressure
FPG: Fasting plasma glucose
2-h PG: 2h post-challenge plasma glucose
HDL: High-density lipoprotein
LDL: Low-density lipoprotein
LTPA: Leisure time physical activity
SD: Standard deviation
IQ: Interquartile
HR: Hazard ratio
CI: Confidence interval
LRC: Lipid Research Clinic
MET: Metabolic equivalents
